# Unilateral pectineal suspension with mesh as a rescue procedure when sacrocolpopexy is not feasible: a case series

**DOI:** 10.1093/jscr/rjaf1094

**Published:** 2026-01-20

**Authors:** Leandro Nobrega, Jerome Mathis, Dimitrios Bolovis, Cosima Brucker, Caroline Eggemann

**Affiliations:** Department of Obstetrics and Gynecology, Spitalzentrum Biel, Vogelsang 84, 2501 Biel/Bienne, Switzerland; Department of Obstetrics and Gynecology, Spitalzentrum Biel, Vogelsang 84, 2501 Biel/Bienne, Switzerland; University Women’s Hospital, Paracelsus Medical University, Prof.-Ernst-Nathan-Straße 1, 90419 Nuremberg, Germany; Paracelsus Medical University, Strubergasse 21, 5020 Salzburg, Austria; University Women’s Hospital, Paracelsus Medical University, Prof.-Ernst-Nathan-Straße 1, 90419 Nuremberg, Germany; Paracelsus Medical University, Strubergasse 21, 5020 Salzburg, Austria; Department of Obstetrics and Gynecology, Spitalzentrum Biel, Vogelsang 84, 2501 Biel/Bienne, Switzerland

**Keywords:** pelvic organ prolapse, sacrocolpopexy, unilateral pectineal suspension, mesh, laparoscopy

## Abstract

Laparoscopic sacrocolpopexy (SCP) is the gold standard for apical prolapse, but presacral dissection may be unsafe or infeasible due to anatomical or surgical barriers. In such cases, alternative fixation methods are needed. We report three elderly women with advanced apical prolapse in whom robotic SCP was converted intraoperatively: one due to redundant sigmoid and bulky presacral vessels, one due to a fibrotic promontory with adherent vessels from prior spinal surgery, and one due to bowel redundancy and adhesions. All underwent unilateral pectineal suspension (UPS) with a lightweight mesh anchored to the right iliopectineal ligament when promontofixation was not feasible. UPS provided stable apical support and functional improvement in all cases. At follow-up, one showed mild asymptomatic mid-compartment descent, one had improved bladder emptying with persistent OAB, and one had excellent correction with mild residual stress incontinence. UPS with mesh is a safe, effective intraoperative alternative when SCP is not feasible.

## Introduction

Apical pelvic organ prolapse (POP)—either uterine descent or post-hysterectomy vaginal vault prolapse—is common in older women. In a large cross-sectional study of elderly women (mean age 68 years), 25% had apical prolapse with the leading edge at or below the hymen, and prevalence reached 63% when stage II or greater by POP-Q criteria was considered [1]. Following hysterectomy, the risk of apical prolapse increases substantially; a cohort showed a 60% higher risk of subsequent prolapse surgery compared with women without hysterectomy [2]. These conditions compromise urinary, bowel, and sexual function and negatively affect quality of life, underscoring the need for durable apical support [3, 4].

Laparoscopic sacrocolpopexy (SCP) is considered the gold standard for apical suspension, with reported anatomic success rates >90% in the short and medium term and remaining > 80% at long-term follow-up [5, 6]. However, SCP requires dissection at the sacral promontory, carrying risks of vascular, bowel, or neural injury, as well as mesh-related complications [7]. In some patients, obesity, redundant sigmoid colon, adhesions, or vascular anomalies render safe promontory access technically difficult or even impossible [8]. In such scenarios, alternative procedures have been described, including laparoscopic pectopexy, high uterosacral ligament suspension, hysteropexy, and lateral suspension [9, 10].

Unilateral pectineal suspension (UPS) is an emerging, minimally invasive technique that anchors the vaginal vault or cervix unilaterally to the pectineal ligament. It was originally described as a standardized robotic, mesh-free procedure with uterus preservation and restoration of the physiologic vaginal axis [11]. Retrospective data suggest good short-term outcomes and safety [12], though evidence remains limited and most experience derives from bilateral pectopexy. To our knowledge, there are no prior reports of UPS performed with a synthetic mesh arm rather than suture-only fixation. We therefore report three cases in which laparoscopic SCP was converted due to hazardous promontory access, and apical support was achieved through unilateral pectineal suspension using a mesh.

## Case series

### Case 1

**Figure 1:**
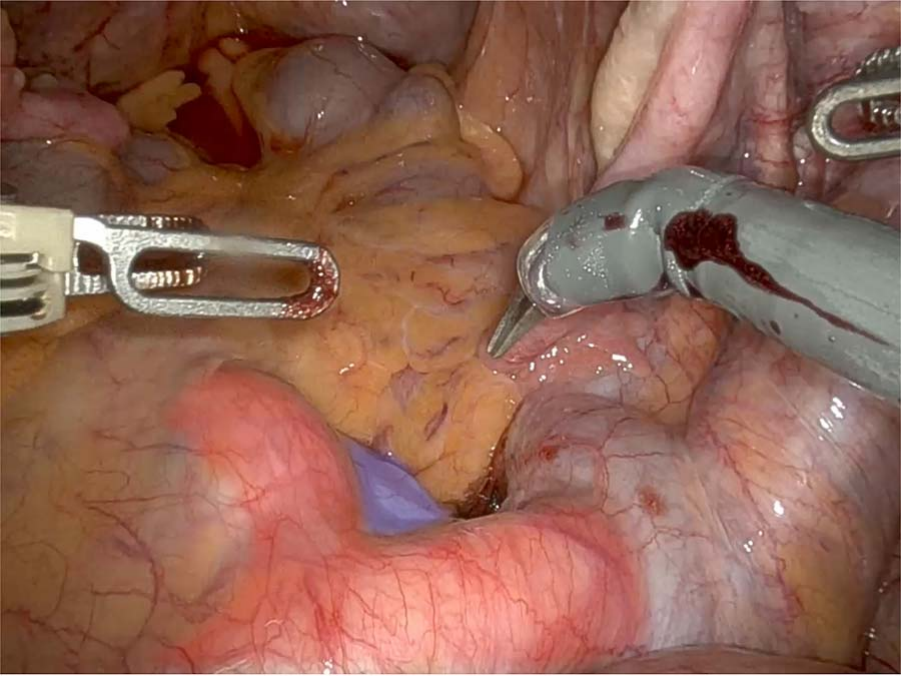
Anterior region to the promontory (in blue) showing varicosity of the sacral venous plexus, with tortuosity and ectasia of the middle sacral vein, positioned between the left and right common iliac arteries (in red).

An 81-year-old woman with POP-Q stage IV apical prolapse underwent initiation of robotic sacrocolpopexy, which was aborted intraoperatively due to redundant sigmoid colon and a dense presacral vascular distribution rendering promontory dissection unsafe (Fig. 1).

**Figure 2:**
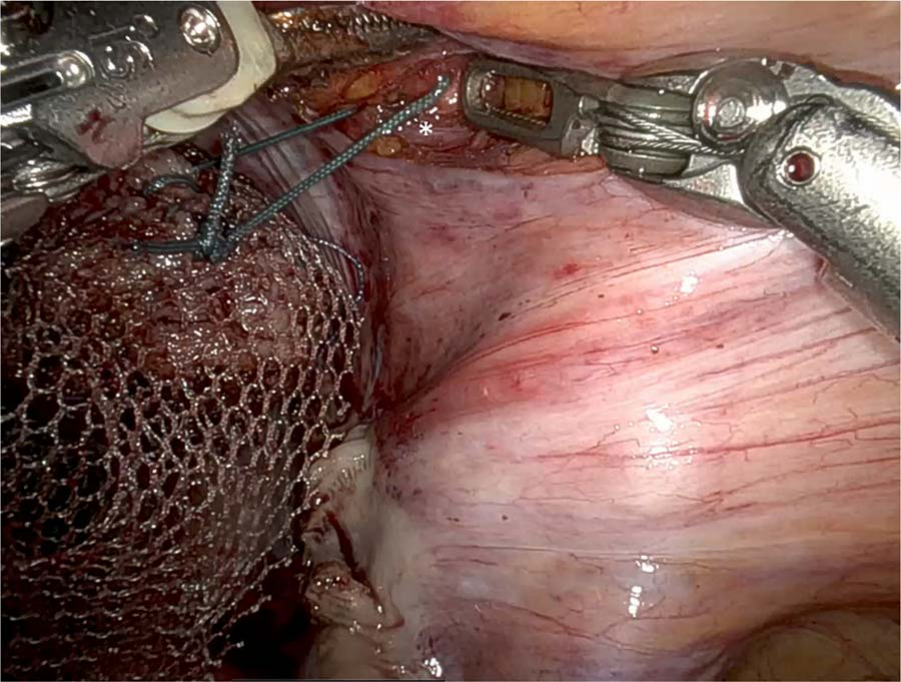
Adapted UPS technique with mesh fixation at the vaginal apex to the right iliopectineal ligament (^*^), using Ethibond® 2.

UPS was performed with a macroporous lightweight mesh (Artisyn®). The distal mesh end was secured to the vaginal apex, and the proximal arm anchored to the right iliopectineal ligament, followed by reperitonealization (Fig. 2).

**Postoperative course:** The patient recovered without complications. Early postoperative examination confirmed good apical suspension with restoration of a physiologic vaginal axis. At 18-month follow-up, clinical examination (documented in POP-Q and images) showed stable apical support. A mild, asymptomatic descent in the mid-compartment was observed but did not require reintervention. The patient reported satisfaction and absence of bulging symptoms.

### Case 2

A 70-year-old woman, with history of previous hysterectomy and lumbar spine surgery, presented with stage II–III apical prolapse (POP-Q C –2 to 0). She complained of stress urinary incontinence and constipation.

**Figure 3:**
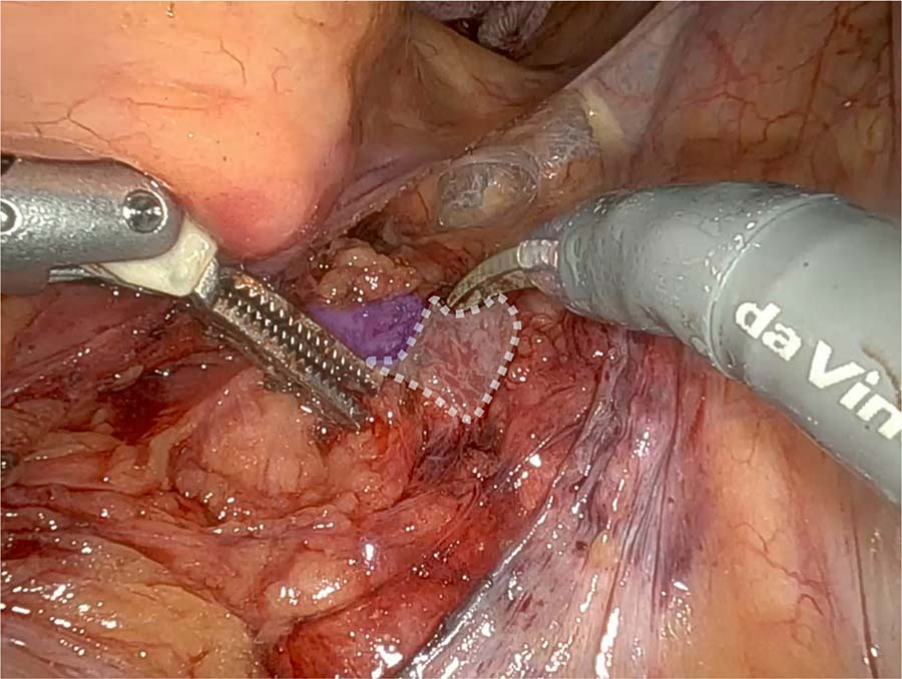
Promontory region showing evidence of surgical cement after spinal surgery (dashed line), located immediately adjacent to the middle sacral vessels (in blue).

**Figure 4:**
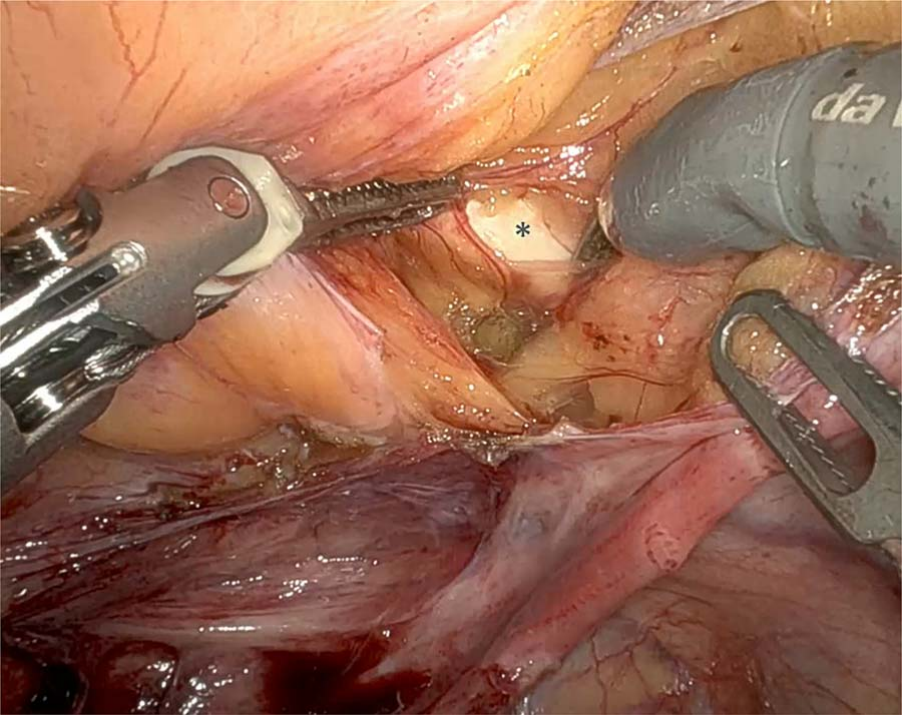
Dissection of the right iliopectineal ligament (^*^) following the UPS technique.

**Figure 5:**
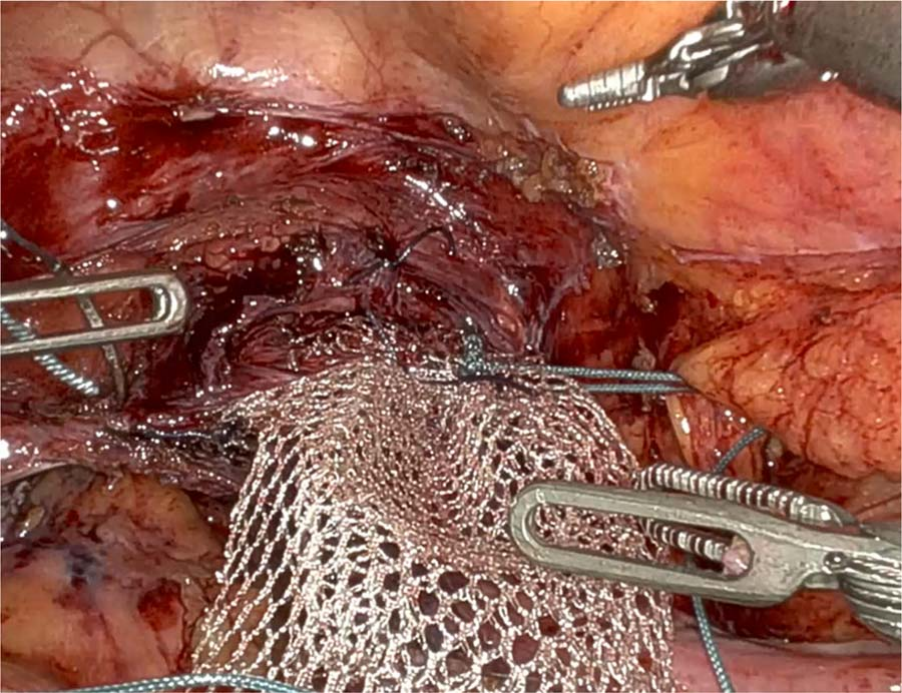
Mesh fixation at the vaginal apex to the right iliopectineal ligament, using Ethibond® 2, representing an adaptation of the original UPS technique.

At surgery, robotic SCP was abandoned due to a fibrous solid structure covering the promontory, with adherent vessels and suspected cement from previous spinal surgery, making safe dissection impossible (Fig. 3) A UPS with PelviGYNious mesh was performed instead, anchoring the vaginal vault to the right pectineal ligament (Figs 4 and 5).

**Postoperative course**: Good recovery. At short follow-up, she had excellent anatomical correction and marked functional improvement. Mild persistent stress incontinence remained, but bowel function normalized and she reported a substantial gain in daily activities.

### Case 3

A 79-year-old woman with Parkinson’s disease presented with POP-Q stage IV apical prolapse and incomplete bladder emptying. Robotic SCP was abandoned intraoperatively due to adhesions and bowel redundancy.

UPS with a synthetic mesh (PelviGYNious®) was performed, anchoring the vaginal cuff to the right iliopectineal ligament. The mesh was tension-free and reperitonealized.

**Postoperative course:** On early follow-up, there was complete correction of apical prolapse with good vaginal axis restoration. The patient reported significant improvement in bladder emptying. At medium-term follow-up, apical support remained stable. Symptoms of overactive bladder persisted but were managed pharmacologically. No mesh exposure or other complications were noted.

## Discussion

Several laparoscopic techniques have been developed to address apical prolapse. SCP remains the gold standard due to its well-documented long-term durability, but it is not universally feasible. Alternatives include laparoscopic pectopexy, in which the apex is suspended bilaterally to the iliopectineal ligaments; laparoscopic lateral suspension (Dubuisson technique), which fixes the apex to the anterior abdominal wall; high uterosacral ligament suspension, a mesh-free option performed laparoscopically; and different variants of hysteropexy in women desiring uterine preservation. Each of these procedures has shown efficacy in apical support, with differing complication profiles and technical demands [9, 10, 13–19].

Within this landscape, UPS has emerged only recently. Originally described by Bolovis and Brucker as a robotic, suture-only, mesh-free procedure anchoring the vaginal vault or cervix to the pectineal ligament [11], UPS is still supported by very limited evidence from small retrospective series and short-term follow-up [12]. To date, no published reports have specifically described UPS using a synthetic mesh arm, making this modification both novel and clinically relevant.

Laparoscopic SCP continues to be the reference procedure, delivering durable anatomic correction and high patient satisfaction [5, 6, 20, 21]. However, SCP requires presacral dissection, with recognized risks of vascular, bowel, and neural injury, as well as late mesh-related complications [7, 22, 23]. Moreover, in certain patients, safe access to the promontory may be technically unachievable, as in cases of obesity, redundant bowel, vascular anomalies, adhesions, or prior spinal surgery [8, 24, 25]. These barriers were precisely encountered in our three patients, prompting abandonment of SCP and adaptation to UPS with mesh.

Laparoscopic pectopexy is the most widely studied alternative, with randomized and observational data showing anatomic and functional outcomes comparable to SCP, but with shorter operative time, less blood loss, and lower rates of defecatory dysfunction [13–16]. Lateral suspension (Dubuisson technique) similarly achieves high apical support rates, with randomized trials confirming equivalence to SCP in the short term, while avoiding presacral dissection and reducing technical complexity [17, 18]. High uterosacral ligament suspension provides a mesh-free approach, attractive in selected patients, but carries higher recurrence risk in advanced multicompartment prolapse compared with SCP [19].

UPS offers several potential advantages, including technical simplicity, avoidance of presacral corridors, and applicability in frail or multimorbid patients. Retrospective data suggest anatomic success above 90% at short-term follow-up [12]. However, evidence remains scarce, limited to small series with retrospective design and lack of long-term outcomes.

Our modification—UPS with a lightweight mesh arm—is, to our knowledge, the first description of such an approach. It preserves the minimally invasive rationale of UPS while broadening the apical fixation surface, mimicking the stability of mesh-based reconstructions. In all three patients, this was employed as a rescue strategy when SCP was unsafe: Case 1 (redundant bowel and bulky presacral vessels), Case 2 (fibrotic promontory and adherent vessels likely due to prior spinal instrumentation) and Case 3 (bowel redundancy and adhesions). Outcomes were encouraging, with good anatomic support, functional improvement, and only mild residual or recurrent symptoms at up to 18 months of follow-up.

Two of the three cases described were illustrated to provide a visual representation of the technique. The third case was not illustrated due to technical issues with image acquisition during the procedure, but the clinical data and outcomes are adequately documented.

### Advantages of UPS

Compared with SCP and other lateral techniques, UPS offers several advantages. First, it avoids presacral dissection, thereby eliminating risks of catastrophic vascular injury, sympathetic plexus damage, and bowel handling required for promontory exposure. This makes UPS particularly useful in patients with obesity, redundant sigmoid colon, adhesions, or altered presacral anatomy [24, 25]. Second, UPS is a technically straightforward and time-efficient procedure, involving a single anchoring point that can be accessed without complex retroperitoneal dissection. Third, it requires minimal mesh material—in our adaptation, only a single lightweight arm—potentially lowering the risk of mesh-related morbidity compared with full bilateral constructs. Fourth, UPS provides centralized apical support while preserving lateral compartments, maintaining vaginal axis and functionality without excessive tension. Fifth, the technique is versatile and easily combined with concomitant procedures such as anterior or posterior repair or anti-incontinence surgery. Finally, its simplicity makes it suitable for frail or multimorbid patients, in whom shorter operative time and lower surgical stress are desirable [12].

In our series, these advantages were reflected in practice: UPS with mesh enabled safe apical suspension in patients where SCP was contraindicated intraoperatively, yielded stable anatomic results at up to 18 months, improved voiding function in one patient, and avoided major perioperative morbidity. While limitations remain—especially the absence of long-term data and the need to monitor for mesh-related complications—the technique demonstrates a pragmatic balance between efficacy and safety.

UPS with mesh offers a technically straightforward, safe, and effective solution when SCP is not feasible due to challenging anatomical factors, such as inaccessible promontory anatomy, obesity, prior spinal surgery, or extensive adhesions. Our series demonstrates that UPS can provide durable anatomical support and meaningful functional improvement, with no major morbidity up to 18 months of follow-up. These early results highlight the potential of UPS as a complementary tool in the management of apical prolapse, especially in patients with complex pelvic anatomy. However, while promising, the long-term durability of UPS and the risk of mesh-related complications remain uncertain. To validate these encouraging short- and mid-term outcomes, further research, including larger, multi-center, and diverse population studies, is warranted to assess its broader applicability and determine whether UPS with mesh can become a standard option in the surgical armamentarium for apical prolapse.

## References

[1] Nygaard I, Bradley C, Brandt D. Pelvic organ prolapse in older women: prevalence and risk factors. Obstet Gynecol 2004;104:489–97. 10.1097/01.AOG.0000136100.10818.d8 15339758

[2] Husby KR, Gradel KO, Klarskov N. Pelvic organ prolapse following hysterectomy on benign indication: a nationwide, nulliparous cohort study. Am J Obstet Gynecol 2022;226:386.e1–9. 10.1016/j.ajog.2021.10.021 34688595

[3] Fatton B, de Tayrac R, Letouzey V, et al. Pelvic organ prolapse and sexual function. Nat Rev Urol 2020;17:373–90. 10.1038/s41585-020-0334-8 32555435

[4] van der Vaart LR, Vollebregt A, Milani AL, et al. Effect of pessary vs surgery on patient-reported improvement in patients with symptomatic pelvic organ prolapse: a randomized clinical trial. JAMA 2022;328:2312–23. 10.1001/jama.2022.22385 36538310 PMC9857016

[5] Claerhout F, De Ridder D, Roovers JP, et al. Medium-term anatomic and functional results of laparoscopic sacrocolpopexy beyond the learning curve. Eur Urol 2009;55:1459–68. 10.1016/j.eururo.2008.12.008 19111382

[6] Sarlos D, Kots L, Ryu G, et al. Long-term follow-up of laparoscopic sacrocolpopexy. Int Urogynecol J 2014;25:1207–12. 10.1007/s00192-014-2369-y 24700356

[7] Haouari MA, Boulay-Coletta I, Khatri G, et al. Complications of mesh sacrocolpopexy and rectopexy: imaging review. Radiographics. 2023;43:e220137. 10.1148/rg.220137 36701247

[8] Capmas P, Suarthana E, Larouche M. Conversion rate of laparoscopic or robotic to open sacrocolpopexy: are there associated factors and complications? Int Urogynecol J 2021;32:2249–56. 10.1007/s00192-020-04570-4 33104825

[9] Chuang FC, Chou YM, Wu LY, et al. Laparoscopic pectopexy: the learning curve and comparison with laparoscopic sacrocolpopexy. Int Urogynecol J 2022;33:1949–56. 10.1007/s00192-021-04934-4 34406417 PMC9270277

[10] Larouche M, Belzile E, Geoffrion R. Surgical management of symptomatic apical pelvic organ prolapse: a systematic review and meta-analysis. Obstet Gynecol 2021;137:1061–73. 10.1097/AOG.0000000000004393 33957652

[11] Bolovis DI, Brucker CVM. Unilateral pectineal suspension - a new surgical approach for apical correction of pelvic organ prolapse. Facts Views Vis Obgyn 2022;14:177–81. 10.52054/FVVO.14.2.015 35781115 PMC10191708

[12] Bolovis DI, Schreibmayer M, Hitzl W, et al. Retrospective analysis of apical prolapse correction by unilateral pectineal suspension: perioperative and short-term results. Int Urogynecol J 2023;34:1877–84. 10.1007/s00192-023-05479-4 36786854 PMC10415474

[13] Lin Y, Liu JJ, Fang K, et al. Pectopexy compared with sacrocolpopexy for the treatment of pelvic organ prolapse: a systematic review and meta-analysis of clinical outcomes. Eur J Obstet Gynecol Reprod Biol 2025;312:114091. 10.1016/j.ejogrb.2025.114091 40494173

[14] Yang Y, Li Z, Si K, et al. Effectiveness of laparoscopic pectopexy for pelvic organ prolapse compared with laparoscopic sacrocolpopexy. J Minim Invasive Gynecol 2023;30:833–40.e2. 10.1016/j.jmig.2023.06.011 37369345

[15] Noé KG, Schiermeier S, Alkatout I, et al. Laparoscopic pectopexy: a prospective, randomized, comparative clinical trial of standard laparoscopic sacral colpocervicopexy with the new laparoscopic pectopexy—postoperative results and intermediate-term follow-up. J Endourol 2015;29:210–5. 10.1089/end.2014.0413 25350228 PMC4313410

[16] Xiao T, Du J, Geng J, et al. Meta-analysis of the comparison of laparoscopic pectopexy and laparoscopic sacrocolpopexy in the treatment of pelvic organ prolapse. Int J Gynaecol Obstet 2025;168:978–86. 10.1002/ijgo.15954 39441550

[17] Malanowska-Jarema E, Starczewski A, Melnyk M, et al. A randomized clinical trial comparing Dubuisson laparoscopic lateral suspension with laparoscopic sacropexy for pelvic organ prolapse: short-term results. J Clin Med 2024;13:1348. 10.3390/jcm13051348 38592190 PMC10931691

[18] Campagna G, Vacca L, Panico G, et al. Laparoscopic lateral suspension for pelvic organ prolapse: a systematic literature review. Eur J Obstet Gynecol Reprod Biol 2021;264:318–29. 10.1016/j.ejogrb.2021.07.044 34364019

[19] Guan Y, Zhang K, Han J, et al. Midterm comparison of laparoscopic high uterosacral ligament suspension and sacrocolpopexy in the treatment of moderate to severe apical prolapse. Int Urogynecol J 2023;34:2501–6. 10.1007/s00192-023-05552-y 37222736

[20] Pacquée S, Nawapun K, Claerhout F, et al. Long-term assessment of a prospective cohort of patients undergoing laparoscopic sacrocolpopexy. Obstet Gynecol 2019;134:323–32. 10.1097/AOG.0000000000003380 31306334

[21] Orhan A, Ozerkan K, Vuruskan H, et al. Long-term follow-up of laparoscopic sacrocolpopexy: comparison of two different techniques used in urology and gynecology. Int Urogynecol J 2019;30:623–32. 10.1007/s00192-018-03858-w 30627828

[22] Thomas TN, Davidson ERW, Lampert EJ, et al. Long-term pelvic organ prolapse recurrence and mesh exposure following sacrocolpopexy. Int Urogynecol J 2020;31:1763–70. 10.1007/s00192-020-04291-8 32253489

[23] Page AS, Cattani L, Pacquée S, et al. Long-term data on graft-related complications after sacrocolpopexy with lightweight compared with heavier-weight mesh. Obstet Gynecol 2023;141:189–98. 10.1097/AOG.0000000000005021 36701619

[24] Wen Q, Zhao Z, Wen J, et al. Impact of obesity on operative complications and outcome after sacrocolpopexy: a systematic review and meta-analysis. Eur J Obstet Gynecol Reprod Biol 2021;258:309–16. 10.1016/j.ejogrb.2021.01.032 33498005

[25] Giraudet G, Protat A, Cosson M. The anatomy of the sacral promontory: how to avoid complications of the sacrocolpopexy procedure. Am J Obstet Gynecol 2018;218:457.e1–3. 10.1016/j.ajog.2017.12.236 29305252

